# Paler within the city: Carotenoid‐based colouration covaries negatively with the proportion of impervious surface in great tit (*Parus major*) territories across Europe

**DOI:** 10.1111/1365-2656.70355

**Published:** 2026-09-18

**Authors:** Nicolas Bekka, Lisa Sandmeyer, Nitha Chardine, Marcel Eens, Tapio Eeva, Arnaud Gregoire, Andrea S. Grunst, Melissa L. Grunst, Caroline Isaksson, Katarzyna Janas, Pablo Salmón, Marta Szulkin, Clotilde Biard

**Affiliations:** ^1^ Institut d'écologie et des Sciences de L'environnement de Paris, IEES, Sorbonne Université, Université Paris Cité, Univ Paris Est Créteil, CNRS, IRD, INRAE Paris France; ^2^ Centre d'Ecologie Fonctionnelle et Evolutive, Univ Montpellier, CNRS, EPHE, IRD Montpellier France; ^3^ Department of Biology, Behavioural Ecology and Ecophysiology Group University of Antwerp Wilrijk Belgium; ^4^ Department of Biology University of Turku Turku Finland; ^5^ Department of Biology Indiana State University Terre Haute Indiana USA; ^6^ Department of Biology Lund University Lund Sweden; ^7^ Museum and Institute of Zoology Polish Academy of Sciences Warsaw Poland; ^8^ Institute of Environmental Sciences Jagiellonian University Kraków Poland; ^9^ Institute of Avian Research “Vogelwarte Helgoland” Wilhelmshaven Germany; ^10^ Faculty of Biology, Biological and Chemical Research Centre, Institute of Evolutionary Biology University of Warsaw Warsaw Poland

**Keywords:** carotenoid‐based plumage colouration, great tit *Parus major*, impervious surface, phenotypic variation, remote sensing, urban ecology, urban evolution, urbanisation

## Abstract

1. Urbanisation alters resource availability and exposes wildlife to novel environmental stressors, potentially driving phenotypic divergence. Carotenoid‐based plumage colouration is a condition‐dependent trait linked to dietary carotenoid acquisition and physiological allocation trade‐offs. Although urban populations are often reported to exhibit paler carotenoid colouration than rural populations, these patterns have rarely been tested along continuous fine‐scale urbanisation gradients at a continental scale.
2. We investigated how reflectance parameters of the yellow breast plumage of adult Great Tit (*Parus major*) covary with territory‐scale local imperviousness, used here as a broad descriptor of urban habitat structure, across six European cities. Using 838 adult observations spanning 2005–2024 and sub‐meter‐resolution satellite imagery, we quantified the proportion of impervious surface within 100 m of nest boxes and related this metric to feather carotenoid chroma, brightness and UV chroma.
3. Carotenoid chroma declined significantly with increasing impervious surface cover within breeding territories across Europe, corresponding to an expected decrease of 0.053 chroma units across the observed 0%–88% imperviousness gradient. This relationship remained robust across bootstrap resampling, leave‐one‐city‐out analyses and models controlling for among‐city variation in mean carotenoid chroma. Older individuals exhibited higher carotenoid chroma than yearlings, while males tended to be more chromatic and brighter than females.
4. Impervious surface cover was not associated with feather brightness or UV chroma, indicating that urban habitat structure is linked specifically to carotenoid pigment deposition rather than feather structural properties and overall quality. Because carotenoid‐based colouration depends on dietary intake, higher levels of impervious cover and reduced vegetation may reflect lower availability of carotenoid‐rich resources in urban territories, although imperviousness should be interpreted as a multifactorial habitat descriptor rather than a direct mechanistic proxy.
5. Our study provides the first continent‐scale evidence that carotenoid‐based colouration declines along a continuous urbanisation gradient. These findings identify plumage carotenoid chroma as a phenotypic correlate of local imperviousness within European cities and highlight the importance of fine‐scale habitat characterisation for understanding phenotypic responses to urbanisation. Future studies should test the mechanistic links between territory‐scale imperviousness, diet composition, pollutant exposure, physiological condition and fitness.

## Graphical abstract

Across six European cities, great tits in more impervious territories had paler carotenoid‐based plumage, but not lower brightness or UV chroma. This suggests that urban habitat imperviousness is associated specifically with lower carotenoid pigment deposition, rather than broad changes in feather reflectance or structural quality.

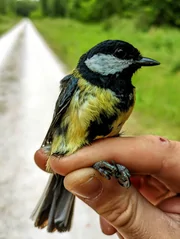

## 1 INTRODUCTION

Urbanisation transforms natural landscapes through fragmentation and land‐use change, creating mosaics of built environments where impervious surfaces and artificial green spaces increasingly replace natural habitats (Antrop, 2004; Grimm et al., 2008). These changes impose novel environmental pressures on wildlife, driving shifts in biodiversity and producing phenotypic divergences between urban and rural populations, which are linked to alterations in landscape composition, biogeochemical cycles and biotic interactions (Alberti et al., 2017). Insects, for instance, often show altered community composition, abundance and phenology in cities (Dale & Frank, 2018), with expected cascading effects on higher trophic levels. In insectivorous and omnivorous birds, one of the most studied taxa in urban ecology (Collins et al., 2021), individuals living in urban populations also reproduce earlier, produce smaller clutches, and have lower body mass than rural counterparts (Caizergues et al., 2018; Chamberlain et al., 2009; Meillère et al., 2015).

Bird plumage ornamentation also frequently differs between urban and rural populations, showing three non‐exclusive main patterns: increased melanism, reduced sexual ornamentation and carotenoid‐based colouration (Cronin et al., 2022; Janas et al., 2024; Leveau, 2021). As examples, kestrel nestlings *Falco tinnunculus* (Sumasgutner et al., 2018), house finches *Haemorhous mexicanus* (Giraudeau et al., 2015) and great tits *Parus major* (Bekka et al., 2025; Eeva et al., 1998; Janas et al., 2026; Salmón et al., 2023), display less chromatic carotenoid‐based colours (i.e. with fewer pigments deposited) in urbanised areas, suggesting that urbanisation imposes environmental constraints affecting carotenoid‐based colouration (Faivre et al., 2003; Isaksson et al., 2005; Peneaux et al., 2021) as habitat structure and resources change. There are several hypotheses commonly used to explain such pattern.

First, urbanisation may reduce carotenoid‐based colouration through food limitation and alteration. Carotenoids cannot be synthesised by vertebrates, which acquire them exclusively from dietary sources (Hill, 1992). Urban areas often lack diverse native plant species and alter the associated arthropod communities (Chatelain et al., 2023; Jensen et al., 2022), lowering the availability of carotenoid‐rich prey such as caterpillars (Eeva et al., 2005) with evidence for diet shifts in urban areas (Chatelain et al., 2023, 2026; Eeva et al., 2005; Pagani‐Núñez et al., 2014; Senar et al., 2021; Sinkovics et al., 2021). In addition, common urban trees tend to have lower carotenoid content, leading to arthropod prey with reduced carotenoid concentrations (Isaksson, 2009; Isaksson & Andersson, 2007). While anthropogenic food sources such as waste and bird feeders are abundant in cities (Oro et al., 2013; Plaza & Lambertucci, 2017), they differ markedly from the carotenoid‐rich invertebrate prey that dominate natural tit diets during the breeding season (Eeva et al., 2009; Isaksson & Andersson, 2007). Dietary limitations in urban areas can therefore reduce feather pigmentation, especially in species with specialised diets (Eeva et al., 2009; McDonnell & Hahs, 2015).

In addition to loss of green areas that can impact diet, urban environments expose avian wildlife to multiple stressors such as chemical pollutants, noise and artificial light (Dominoni et al., 2013; Halfwerk et al., 2011; Isaksson, 2015), which increase oxidative stress and disrupt functions such as homeostasis and reproduction (Grimm et al., 2008; Isaksson, 2015). Urban environments also are hotspots for pathogen transmission through elevated contact rates (Bradley & Altizer, 2007; Wright & Gompper, 2005). As carotenoids are involved in antioxidant and immune functions (Surai, 2002; Svensson & Wong, 2011; Weaver et al., 2018), these increased physiological demands in urban settings may further constrain the deposition of carotenoids to plumage colouration through an allocation trade‐off with physiological functions (reviewed in Møller et al., 2000; but see Hill et al., 2023; Koch & Hill, 2018; Weaver et al., 2018 for critical reviews of tests of this hypothesis).

Alongside these effects on carotenoid acquisition and allocation during moult, which are the main hypotheses explored in the literature, plumage colour can change over time between moults (Delhey et al., 2010), through feather wear and environmental degradation that could be enhanced by urban habitat characteristics such as the deposition of pollutants and airborne particles on feathers (Surmacki et al., 2023), greater bacterial loads (Sotnychuk et al., 2020) or greater abrasion on mineral surfaces (Surmacki et al., 2011). The observed paleness of urban birds as compared to rural birds may also arise from several non‐exclusive ecological and evolutionary pathways as fragmented urban habitats may induce movement constraints and reduced dispersal in urban areas (Johnson & Munshi‐South, 2017; Markowski et al., 2021). Moreover, if paler urban birds are not randomly distributed across urban habitats, we can conjecture phenotype‐habitat matching through competitive sorting processes (Fokkema et al., 2021) and/or selection towards paleness and correlated traits in urbanised habitats (Bekka et al., 2025). Because yellow feather colouration can be described by several complementary reflectance metrics, comparing carotenoid chroma with brightness and UV chroma, which are commonly interpreted as measures of feather structural quality (Jacot et al., 2010; Shawkey & Hill, 2005), may help distinguish among mechanisms underlying associations between plumage colouration and urban habitat structure. An association restricted to carotenoid chroma would be more consistent with processes affecting carotenoid acquisition or deposition during feather growth. In contrast, concordant associations across carotenoid chroma, brightness and UV chroma would be more consistent with broader alterations in feather properties, including structural quality or post‐moult degradation through wear, abrasion, bacterial degradation or pollutant deposition.

Although urban–rural comparisons are common, phenotypic responses along continuous, fine‐scale urbanisation gradients remain less studied (Szulkin et al., 2020; but see, Giraudeau et al., 2018; Janas et al., 2026). Indeed, some studies compare a single urban site with a nearby rural habitat, thereby overlooking the heterogeneity of urban mosaics (McDonnell & Pickett, 1990; Norton et al., 2016). As a result, categorical urban–rural comparisons pool together urban habitats that strongly differ in structure, limiting inferences about how urbanisation processes influence phenotypic variation. Moreover, reported effect sizes vary markedly among sites in urban–rural comparisons (Salmón et al., 2023), likely reflecting differences in local habitat structure and environmental pressures. To overcome these limitations, replicated studies that test phenotypic responses along continuous urbanisation gradients and across multiple sites are needed (Alberti et al., 2017; Ouyang et al., 2018; Szulkin et al., 2020). Beyond avoiding the oversimplification inherent to urban–rural contrasts, this framework better captures the within‐city heterogeneity and allows us to refine inferences about ecological correlates of phenotypic variation linked to urbanisation. In addition, studying continuous variation across multiple urban areas can help better understand the urban characteristics underlying phenotypic variation in cities. Overall, this may allow us to identify potential parallel eco‐evolutionary processes driven by urbanisation. To do so, high‐resolution satellite imagery offers a valuable and reproducible tool for quantifying urban features but commonly used landscape maps such as Corine Land Cover may fail to capture fine‐scale urban variation (Diaz‐Pacheco & Gutiérrez, 2014). Coarse resolution products (e.g. CORINE Land Cover, 2018) generalise mixed pixels to attribute a single land‐cover class for each cell, smoothing over small objects (CORINE Land Cover, 2018; European Environment Agency, 2019), and thereby obscuring habitat heterogeneity relevant to species with small territories and the detection of fine ecological patterns (Cooper et al., 2020; Grafius et al., 2016; Rioux et al., 2019). Higher‐resolution habitat characterisation can better detect structural variation within urban areas, yet continental‐scale habitat maps at this resolution remain scarce. Developing fine‐scale characterisation of urban habitat structure is therefore crucial for linking local urban features to large‐scale ecological patterns.

Our study aims to fill this gap and apply this approach by combining for the first time continental‐scale replication of urban populations with fine‐scale measures of habitat structure within the breeding territory, thereby testing whether variation in plumage colouration tracks continuous differences in urban habitat structure across Europe. We compiled carotenoid‐based plumage colouration from great tits breeding in six European cities and quantified the proportion of impervious surface within territories, using the highest‐resolution measurements available to date from free Google satellite imagery, that is <1 m. As imperviousness is a generalised index of environmental disturbance, it reflects the lack of vegetation and associated arthropod communities (Raupp et al., 2010) and should therefore effectively capture the scarcity of carotenoid‐rich resources for insectivorous birds. We focused on carotenoid chroma as our main response variable because it is the metric most directly linked to carotenoid pigment deposition (Saks et al., 2003). We additionally examined brightness and UV chroma as complementary descriptors of yellow breast feather reflectance properties. This approach allowed us to determine whether associations between urban‐induced paleness and impervious surface cover were specific to carotenoid deposition or reflected broader variation in feather reflectance characteristics and structural quality (Jacot et al., 2010; Shawkey & Hill, 2005). We hypothesised that carotenoid‐based plumage colouration correlates with local imperviousness at a fine scale across populations, as well as brightness and UV chroma, following a pattern documented in a recent study comparing urban and rural populations (Sandmeyer et al., 2025). We included sex and age as covariates because yellow plumage reflectance in great tits can vary between age and sex classes, although the direction and strength of these differences depend on the colour metric considered (Isaksson et al., 2008; Sandmeyer et al., 2025). Based on previous evidence that older individuals can show more saturated carotenoid‐based yellow colouration, and that males may differ from females in brightness and other reflectance components, we expected older birds and males to show higher values of reflectance for at least some yellow feather colour metrics. We also tested whether the relationship between imperviousness and yellow feather colouration differed between sex and age classes, because previous studies suggest that urban effects on carotenoid‐based colouration in great tits may be age‐ and sex‐dependent, with first‐year birds and males sometimes showing stronger reductions in urban habitats (Salmón et al., 2023; Sandmeyer et al., 2025). We predicted that increasing impervious surface cover within breeding territories will be associated with reduced yellow breast feather colouration and reflectance, primarily through lower carotenoid chroma and secondarily through lower UV chroma and brightness.

## 2 MATERIALS AND METHODS

### 2.1 Study species, sites and data collection

The great tit is a widespread, generalist and omnivorous passerine that breeds territorially in cavities. Individuals first breed at 1 year and have an average lifespan of ~3 years (Bouwhuis et al., 2009). Carotenoid‐based yellow breast plumage in great tits (Partali et al., 1987) is reported to vary with age in urban environments with older birds showing higher carotenoid chroma (Bekka et al., 2025; Salmón et al., 2023; Sandmeyer et al., 2025). Great tit populations are widely monitored across Europe using nest boxes, with similar standardised protocols for capture, measurement and sampling. Here we analysed six urban study systems across Europe (Figure 1): Antwerp (Belgium), Montpellier (France), Paris (France), Malmö (Sweden), Harjavalta (Finland) and Warsaw (Poland). Detailed site‐specific protocols are described for Antwerp (Grunst et al., 2020), Montpellier (Sandmeyer et al., 2025), Paris (Bekka et al., 2025), Malmö (Salmón et al., 2023), Harjavalta (Eeva et al., 2009) and Warsaw (Janas et al., 2026).

**FIGURE 1:**
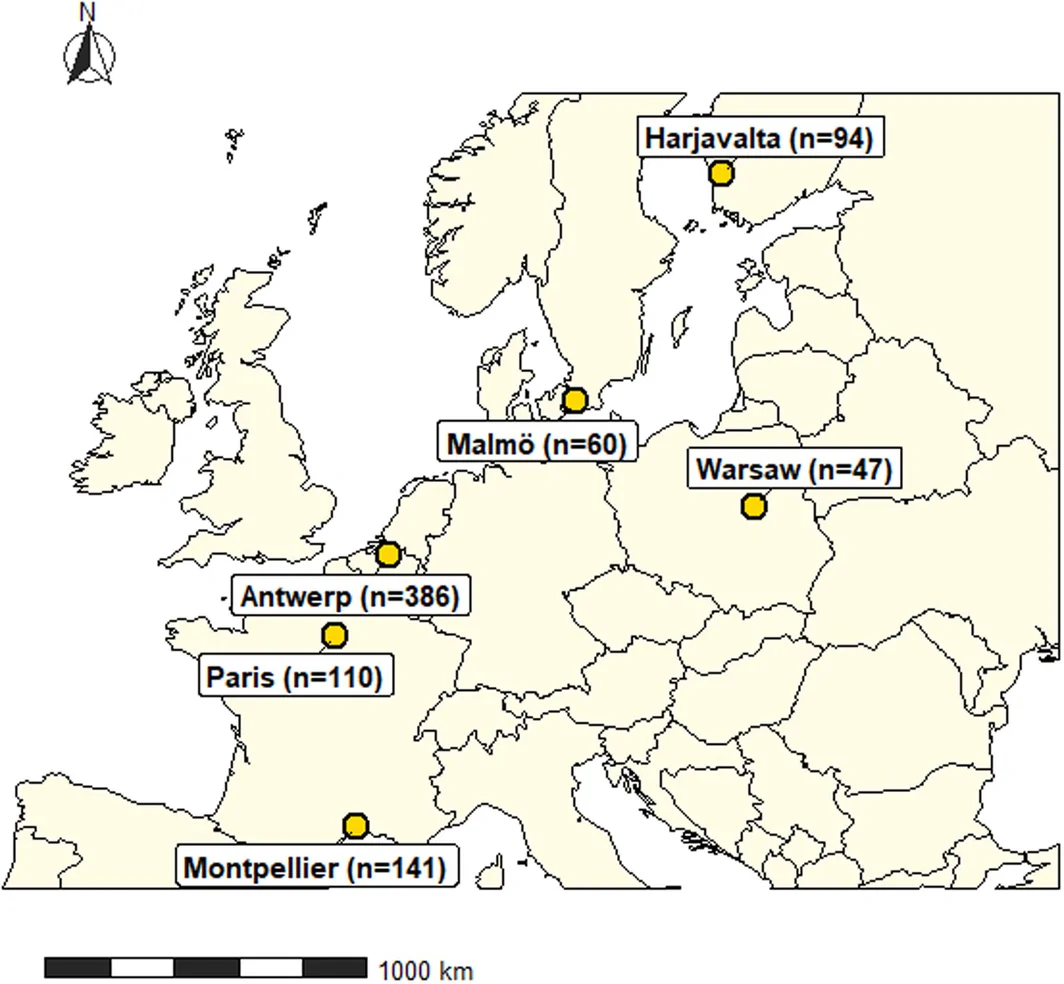
Yellow circles mark the six European great tit (*Parus major*) populations sampled for colour analyses; labels give the number of observation points per city. Country boundaries are shown for reference only.

At each site, adults were captured at nest boxes during the breeding season, 7–14 days after hatching. Sex was determined by the presence of a brood patch in females and plumage characteristics, that is larger black breast stripe of males. Age was assigned following Svensson (1984): one‐year‐old birds were identified by contrast between greater primary coverts and the rest of the wing plumage, while older birds (≥2 years) lacked this contrast. From each bird, 5–10 yellow breast feathers were plucked and stored individually in paper or plastic envelopes in the dark until analysis. In total, we compiled colouration data from 838 observations across six European cities and 8 sampling years, spanning 2005–2024 (Figure 1, detailed sample sizes by city, sex and age class are provided in Table S1). This work conforms to European regulations regarding the use and ethical treatment of wild animals for scientific research and has been conducted under appropriate licences and ethical authorisations. Antwerp: The University of Antwerp's ethical committee approved field methodology (ID 2016‐71). Harjavalta: The data were collected under the licences of the Animal Care & Use Committee of Turku University and Regional Environmental Centre. Malmö: Permission to collect samples was issued by the Local Ethical Committee in Malmö/Lund (permit no. M454 12:1). Montpellier: Permission to collect samples was issued by CRBPO (Centre de Recherches sur la Biologie des Populations d'Oiseaux, Museum National d'Histoire Naturelle, Paris) through Personal Project no 369 and bird ringing licence no 1904. Paris: The data were collected under authorisations no DRIEE‐2012‐31à38, 2019‐DRIEE‐IF/033 and /046, ethical approval no APAFIS#19941‐2019032516275025v5, and bird ringing licence no 1454 and Personal Project no 537 delivered by CRBPO. Warsaw: Permission to collect samples was issued by the Local Ethical Committee nr I for Animal Experimentation in Warsaw (I Lokalna Komisja Etyczna ds. Doświadczeń na Zwierzętach w Warszawie; permit no. 1548P1[KJ1]/2016).

### 2.2 Quantification of impervious surface

To standardise habitat quantification at a biologically relevant scale, we calculated impervious versus vegetation cover within 100 m around each nest box, matching typical great tit territory size (Bouvier et al., 2022) and enabling direct comparison with prior great tits urban territory‐mapping approaches (Chatelain et al., 2021; Janas et al., 2026), using QGIS v.3.34 (QGIS Development Team, 2024). High‐resolution (<1 m per pixel in cities) Google satellite imagery (EPSG:3857) was exported as GeoTIFFs for each buffer and classified into two land‐cover categories (vegetation vs. impervious surface) with the Semi‐Automatic Classification Plugin (SCP; Congedo, 2021). This binary classification was designed to estimate the proportion of impervious surface at the territory scale; under this framework, vegetation cover is the direct complement of impervious surface rather than an independent predictor. For Harjavalta, where feathers were collected in 2005 and 2008, we used historical 2009 orthophotos obtained from the National Land Survey of Finland data service rather than 2024 imagery, which was the closest available match to biological sampling date. For the remaining sites, we retained the most recent high‐resolution Google satellite imagery available in 2024, assuming that impervious surfaces are among the more temporally stable components of urban characteristics, especially in urban cores. Thus, imagery year could not be matched to sampling year at every site, but the strongest temporal mismatch was reduced for Harjavalta. We applied a supervised nearest‐neighbour algorithm using at least 10 training polygons per class for each sub‐site within a city (e.g. each park containing nestboxes), to capture the full range of local spectral variability (e.g. sunlit and shaded pavement, evergreen and deciduous vegetation). For each 100 m radius buffer, misclassifications were corrected by importing OpenStreetMap road and building layers (QuickOSM), buffering roads by 6 m as it visually best matched roads width in our study sites, to overwrite mislabelled pixels as ‘impervious’. The corrected raster was polygonised, clipped to each buffer and visually inspected for residual errors (e.g. shadows). For each buffer, we calculated the total area (m^2^) of impervious and vegetation cover. We then calculated the proportion of impervious surface in territories by dividing the impervious surface area by the total area of the 100 m radius buffer.

### 2.3 Feather colour analysis

Feather colour was quantified by reflectance spectrophotometry. Measurements were taken by four trained observers. Site‐specific protocols, instrumentation (spectrometer, lamp, probe) and calibration routines (dark/white standards) followed published protocols for Paris, Harjavalta and Malmö (Bekka et al., 2025), Montpellier (Sandmeyer et al., 2025), Antwerp (Grunst et al., 2020) and Warsaw (Janas et al., 2026). Briefly, feathers were illuminated at an angle of 90° and reflected light was measured at the same angle with 6–10 measurements per feather stack. For each individual, 5–10 breast feathers were stacked and 6–10 spectra were recorded on randomly selected spots in the yellow tip of the stacked feathers.

Among structurally related and pigmentation related metrics from reflectance spectra, we analysed carotenoid chroma as our main response variable. Because carotenoid chroma is a reflectance metric directly linked to carotenoid pigment deposition (Saks et al., 2003), it maximises comparability with prior urban studies on colouration and is a suitable response variable for testing whether yellow feather colouration covaries with local imperviousness. We additionally analysed brightness and UV chroma as complementary reflectance metrics, to test whether any association with imperviousness was specific to carotenoid chroma or extended more broadly across feather reflectance traits. Carotenoid chroma was calculated with *pavo* R package (Maia et al., 2019) for all measured colour spectra, as the relative difference between maximal (plateau above 500 nm) and minimal (between 445 and 455 nm) reflectance in the visible spectrum: [R700 − R450]/R700 (Peters et al., 2007). Total brightness was calculated as the sum of relative reflectance across the full measured spectral range [300–700] nm, and UV chroma as the proportion of total brightness represented by reflectance in the UV range (from 300 to 400 nm; Maia et al., 2019; Montgomerie, 2006). Average values for the 6 to 10 measures per individual sample were used in subsequent statistical analyses. Carotenoid chroma, brightness and UV chroma repeatability (R) at the bird level was estimated with *rpt*() function from *rptR* package with 95% CIs from 1000 bootstrap resamples (Stoffel et al., 2017). Replicate scans (and multiple feathers, where present) were assigned to the same *Bird* level so that R reflects overall within‐bird consistency across measurements. Repeatability for carotenoid chroma was high at all sites: Antwerp *R* = 0.76 (95% CI = 0.73–0.78), Warsaw *R* = 0.68 (0.56–0.75), Malmö–Harjavalta–Paris combined, as they share the same observer and protocol, *R* = 0.64 (0.60–0.67) and Montpellier *R* = 0.77 (0.72–0.81). Repeatability for total brightness was moderate to high: Antwerp *R* = 0.53 (95% CI = 0.48–0.57), Warsaw *R* = 0.59 (0.46–0.68), Malmö–Harjavalta–Paris combined, as they share the same observer and protocol, *R* = 0.58 (0.53–0.61) and Montpellier *R* = 0.92 (0.90–0.94). Repeatability for UV chroma was moderate to high: Antwerp *R* = 0.81 (95% CI = 0.79–0.83), Warsaw *R* = 0.68 (0.58–0.77), Malmö–Harjavalta–Paris combined, as they share the same observer and protocol, *R* = 0.53 (0.49–0.57) and Montpellier *R* = 0.62 (0.56–0.68). Because repeatability was moderate to high for each colour parameter, average values for the 6 to 10 measures per individual sample were used in subsequent statistical analyses.

### 2.4 Statistical analysis

The analyses were run on R version 4.5.1. The dataset and scripts used for the analyses are archived in a Zenodo repository (Bekka et al., 2026).

We analysed variation in carotenoid chroma, brightness and UV chroma using linear mixed‐effects models. City, nest box, bird identification number and year were fitted as random intercepts to account for site‐ and cohort‐level non‐independence of observations. Main fixed effects included the proportion of impervious surface within 100 m of each nest box, sex (female, male) and age (1 or ≥2 years old). We first fitted a maximal model including interactions between proportion of impervious surface and sex, and between proportion of impervious surface and age, in which proportion of impervious surface was additionally allowed to vary among cities as a random slope. Model simplification used the *step*() function from the *lmerTest* package (Kuznetsova et al., 2017), which sequentially removed unsupported interaction terms and random‐effect components. Models were fitted by REML using *lmerTest*. Fixed‐effect tests used Satterthwaite degrees of freedom. To plot marginal effects, we generated predictions across the proportion of impervious surfaces (0–1 by 0.01) using the *ggpredict*() function from the *ggeffects* package (Lüdecke, 2018), with other covariates held at their reference/observed values as implemented in that package.

To assess slope stability of the relationship between carotenoid chroma and proportion of impervious surface around the nest box, and control for the unequal sample sizes between sites, we performed a stratified bootstrap. For each of 1000 replicates, we picked randomly up to 47 individuals per city, corresponding to the smallest sample size (Warsaw), refitted the supported mixed model and recorded the fixed‐effect slope for proportion of impervious surface. We summarised the bootstrap distribution by its mean, standard deviation, percentile 95% interval [2.5; 97.5%] and the proportion of replicates with a negative slope. A reproducible seed (seed number = 2025) was set prior to resampling.

To assess whether the imperviousness effect was driven by any single site, we performed a leave‐one‐site‐out analysis. We refitted the model six times, each time excluding one city in turn. For each refit, we extracted the fixed‐effect slope for impervious cover (estimate and Wald 95% CI). This analysis tests directional robustness and highlights any sites that, due to larger *x*‐axis range or sample size, might drive observed patterns.

Finally, as a complementary robustness analysis, we centred carotenoid chroma within city (i.e. subtracted the city mean from each observation) and refitted the mixed model with imperviousness, sex and age as fixed effects, and year and bird identification number as random effects, to test whether the association with imperviousness persisted independently of between‐city differences in mean colouration.

## 3 RESULTS

Territories spanned low to very high imperviousness across cities. Montpellier and Paris showed the highest average impervious cover (46% and 38%), with Montpellier and Harjavalta also exhibiting the broadest ranges (up to 88%). Warsaw, Harjavalta and Antwerp were lower on average (22%–29%), with narrower upper tails (Figure 2).

**FIGURE 2:**
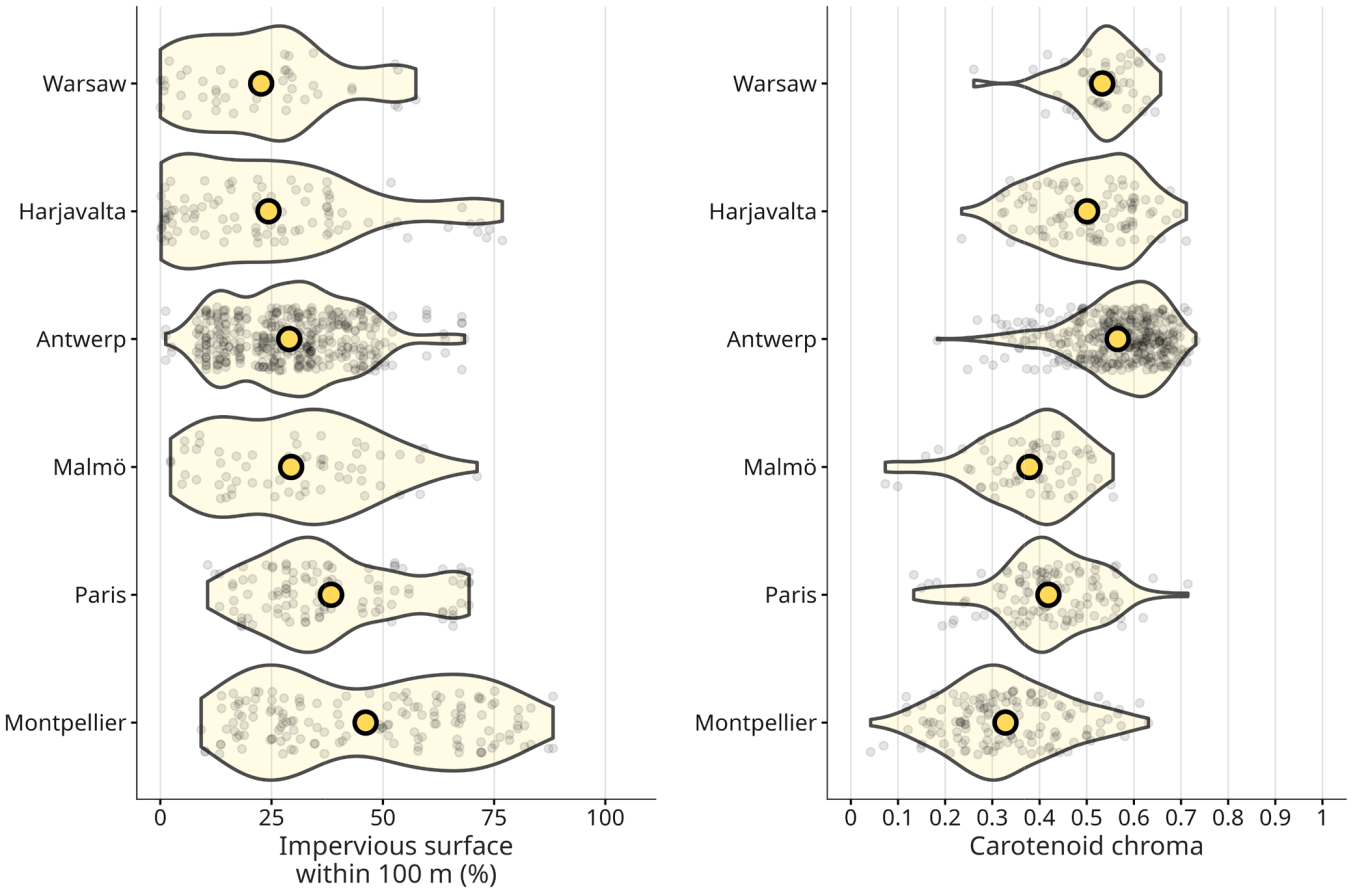
Violin plots of the proportion of impervious surface (%) distribution within 100‐m buffers for each site on the left panel, and carotenoid chroma on the right panel; yellow points indicate means. Means for proportion of impervious surface (range): Antwerp 29% (1–68), Harjavalta 24% (0–88), Malmö 29% (2–71), Warsaw 22% (0–57), Paris 38% (11–69), Montpellier 46% (9–88). Means for carotenoid chroma (range): Antwerp 0.57 (0.18–0.73), Harjavalta 0.50 (0.23–0.71), Malmö 0.38 (0.07–0.56), Warsaw 0.53 (0.26–0.66), Paris 0.42 (0.13–0.71), Montpellier 0.33 (0.04–0.63).

Likelihood ratio tests from the maximal model showed that including random intercepts for bird identification number, city and year improved fit (all *p*‐values <0.01), whereas including a nest box intercept and a random slope for impervious surface by city did not. In the supported model including random intercepts for bird identification number, city and year, interactions between impervious surface and sex or age were not statistically significant (impervious × sex: *F* = 1.46, *p*‐value = 0.23 and impervious × age: *F* = 0.09, *p*‐value = 0.77) and not retained in the final model. The slope of impervious surface was significantly negative (*β* = −0.05 ± 0.02 SE; *t* = −2.65, df = 739.1, *p*‐value <0.01; Figure 3a), corresponding to an expected decline of 0.053 chroma units across the observed 0%–88% imperviousness gradient. Chroma increased with age (*β* = 0.04 ± 0.01; *t* = 6.12, df = 813.9, *p*‐value <0.001) and showed a tendency to be higher in males (*β* = 0.01 ± 0.007; *t* = 1.66, df = 676.18, *p*‐value = 0.09).

**FIGURE 3:**
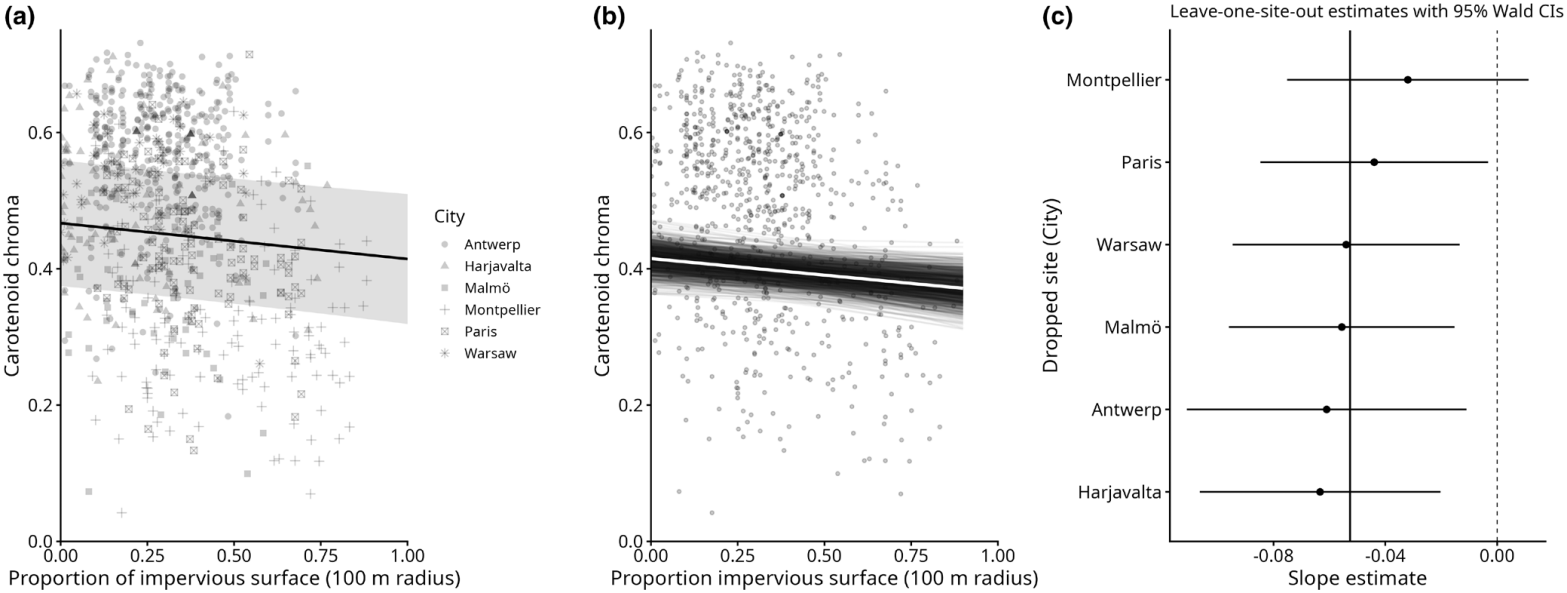
(a) Covariation between carotenoid chroma and proportion of impervious surface in the bird's territory. Points are individual great tit observations (symbols = city; semi‐transparent). The solid line is the marginal effect of impervious cover from a linear mixed model with fixed effects of impervious cover, age and sex, and random intercepts for City, Year and Individual ID; the grey band is the model 95% CI. (b) Thin grey lines show fitted relationships from 1000 stratified bootstrap refits (47 birds per site per replicate); the white line is the bootstrap mean. Across replicates, 99% of slopes were negative. (c) Leave‐one‐site‐out sensitivity of the impervious surface effect. Points are the estimated slopes (*β* for the proportion of impervious surface in bird territory) from the mixed model refit after dropping the site on the *y*‐axis; horizontal bars are 95% Wald CIs (Satterthwaite). The bold black vertical line is the full‐data estimate (*β* ≈ −0.05) and the dashed line denotes zero. Models include fixed effects of age and sex and random intercepts for City, Year and Individual ID.

A stratified bootstrap of 1000 resamples, with 47 individuals randomly picked per city, yielded a mean slope of −0.049 with a 95% CI of [−0.094; −0.005], and 98.9% of bootstrap replicates with a negative slope (Figure 3b). Thus, the direction of the effect was highly consistent across resamples, aligning with the significant mixed‐model estimate.

Leave‐one‐site‐out estimates were negative for every city (range − 0.063 to −0.032; mean slope = −0.052, Figure 3c), and five of six 95% CIs excluded zero. The only case with a CI overlapping zero was when Montpellier was omitted (slope = −0.032 [−0.075; 0.011] 95% CI). This loss of significance likely reflects a reduced x‐axis range rather than a site‐specific anomaly as Montpellier supplies much of the highly urbanised tail, so including it increases slope precision. Overall, the effect's direction and magnitude remained robust to the removal of any single site. To test whether the negative association with imperviousness persisted independently of between‐city differences in mean chroma, we fitted an additional model using carotenoid chroma centred within each city. The effect of impervious surface remained significantly negative (*β* = −0.058 ± 0.019 SE, *t* = −3.00, *p* < 0.01), supporting the statement that the overall pattern was not solely driven by differences in mean colouration among cities.

For the other colouration traits, neither brightness nor UV chroma covaried with proportion of impervious cover (brightness: *β* = 242.61 ± 438.91 SE, *t* = 0.58, *p* = 0.58; UV chroma: *β* = 0.0005 ± 0.006 SE, *t* = 0.08, *p* = 0.93, details in Table S3 for brightness, and in Table S4 for UV chroma). Brightness was higher in males (*β* = 1141.86 ± 158.97 SE, *t* = 7.18, *p* < 0.001) than in females, but neither brightness nor UV chroma varied with age (*t* < 0.42, *p* > 0.68).

## 4 DISCUSSION

This study represents the first continental‐scale assessment of how continuous variation in urban‐associated habitat change is linked with carotenoid‐based colouration within urban areas, combining fine‐scale habitat characterisation of impervious cover with large‐scale replication across Europe. We find that across six European cities, carotenoid chroma declined with increasing impervious cover. Results were concordant across a linear mixed model and a stratified, site‐balanced bootstrap: 99% of bootstrap slopes were negative and the 95% bootstrap CI did not overlap zero, corresponding to a decline of ~0.05 chroma units across the observed 0%–88% imperviousness gradient. A leave‐one‐site‐out analysis also yielded negative slopes for every city. Only when omitting Montpellier did the 95% CI marginally include zero, likely reflecting reduced imperviousness range in the pooled remaining dataset rather than a change in effect. Furthermore, the negative association remained significant when carotenoid chroma was centred within each city, indicating that the overall pattern is not only driven by between‐city differences in mean colouration. Age and sex effects followed expectations for carotenoid chroma only, with older birds being more chromatic and males slightly more chromatic than females, without evidence that the slope describing carotenoid chroma relationship with imperviousness varied over classes. We found no effect of impervious cover on brightness and UV chroma of the yellow breast feathers.

Our findings are consistent with those of Janas et al. (2026), who reported paler carotenoid‐based colouration along a continuous urbanisation gradient across Polish cities, and with other studies showing reduced carotenoid‐based colouration in urban great tits as compared to rural ones (Bekka et al., 2025; Salmón et al., 2023; Sandmeyer et al., 2025). We extend this pattern to a broader European context by showing that, across six cities, birds occupying the most impervious territories within cities are also paler across the continent. We also showed that brightness and UV chroma did not vary with imperviousness. This suggests that the observed association is specific to carotenoid chroma and therefore more linked to the amount of pigment deposited in feathers. Because brightness and UV chroma reflect aspects of feather structural quality (Jacot et al., 2010; Shawkey & Hill, 2005), the absence of an association with impervious cover does not support a broad decline in feather overall reflectance and structural quality in concrete habitats. Furthermore, although impervious cover can be viewed as a generalised descriptor of urban habitat disturbance, it may not capture equally well all environmental factors expected to affect structural components of feather reflectance, such as airborne particles exposure, enhanced abrasion or feather degradation through enhanced parasitic load (Sotnychuk et al., 2020). In comparison, it may more closely reflect habitat characteristics linked to food resource availability, composition and quality, as our pattern indeed accords with the resource constraints hypothesis, where highly built surfaces lack vegetation and associated arthropods, potentially reducing dietary carotenoids (Chatelain et al., 2023, 2026; Eeva et al., 2005).

Although the direction of the association was consistent across analyses, imperviousness explained only a limited fraction of variation in carotenoid chroma. The modest slope therefore suggests that the observed association reflects a multifactorial environmental signal, in which vegetation composition, prey community structure and phenology, anthropogenic food availability and microhabitat characteristics may contribute besides impervious surface. In particular, the vegetation composition may matter as much or more than vegetation amount, and urban stressors such as noise, artificial light and pollutants may furthermore elevate antioxidant demands (Isaksson, 2015) and divert carotenoids from plumage deposition. Impervious surface cover should therefore be interpreted as a broad descriptor of urban habitat structure rather than as a direct proxy for a single mechanism. As feathers measured in year *n* were grown during the post‐breeding moult of year *n*–1, we document a spatial association at breeding between carotenoid chroma and local impervious cover, rather than a direct test of the conditions under which those feathers were produced. This temporal offset precludes causal inference about moult environments, but two points might mitigate this concern: impervious surfaces such as roads and buildings are relatively persistent and long‐lasting features, and adult great tits show high breeding site fidelity (Harvey et al., 1979). However, post‐fledging and seasonal movements in tits may generate mismatches between natal and moulting environments in non‐urban environments (Naef‐Daenzer & Grüebler, 2008; Slagsvold et al., 2013; van Overveld et al., 2011), therefore imperviousness in our study is not a reconstruction of the environment experienced during feather growth. The persistence of the colour‐imperviousness association across the moult‐breed temporal gap may itself be informative. If feather colour simply tracked immediate environmental conditions during moult, we might expect the association with breeding territory to weaken or disappear. Instead, its consistency across cities suggests carotenoid‐based colour reflects a more stable individual‐level trait reflecting long‐term habitat association, making colour a tracker of the bird's broader relationship with its urban environment rather than a snapshot of moult‐time environmental conditions.

Understanding multifactorial mechanisms underlying effects of urbanisation on carotenoid‐based colouration will require spatially and temporally matched data at the territory scale, which were not available across cities and sampling years in the present study. Nonetheless, our approach highlights the value of fine‐scale habitat characterisation when the ecological question is posed at the territory scale. In heterogeneous urban landscapes, lower‐resolution land‐cover products smooth over small habitat patches and underestimate local variation in vegetation and impervious surfaces, altering ecological inference (Grafius et al., 2016; Rioux et al., 2019). Fine‐scale habitat metrics are therefore particularly relevant for species whose breeding ecology is shaped by environmental variation at the scale of individual territories. For this reason, high‐resolution, widely accessible imagery such as Google satellite imagery, may better retain the small‐scale heterogeneity of urban mosaics, which is likely to matter for species such as great tits breeding in relatively small territories. We would therefore recommend this approach when the aim is to quantify local urban habitat structure with high spatial precision in comparative studies spanning cities from different countries or continents, where the use of different regional land‐cover products could reduce methodological homogeneity, and where imagery availability and coverage might not be fully standardised. However, our classification still involves analyst‐dependent decisions, particularly in the choice of training polygons and post‐classification corrections, and temporal matching with biological sampling is not always possible. A related methodological limitation concerns temporal matching between habitat quantification and plumage sampling. Indeed, exact temporal matching remained impossible for most sites. Thus, our approach prioritised fine spatial resolution at the territory scale over full temporal consistency, assuming that impervious surfaces are among the more temporally stable components of urban habitat structure. Coarser but temporally explicit land‐cover products would offer the opposite trade‐off.

Several non‐exclusive mechanisms could generate the observed pattern. (i) Movement constraints: limited natal and reproductive dispersal, and resulting philopatry in fragmented urban landscapes, could cluster individuals within natal or nearby patches. It could produce spatial sorting independent of territory choice, mainly reflecting dietary constraints. Such constraints during early developmental stages are known to influence long‐term ability to acquire and use carotenoids (reviewed in Biard et al., 2009), that might reflect in adulthood during moult events. (ii) Carryover from breeding to moult: conditions experienced during the preceding breeding season, such as exposure to chemical and physical pollution, may elevate carotenoid demands for physiological defence (Dauwe & Eens, 2008; Geens et al., 2009; Isaksson, 2015) and reduce deposition into feathers (but see Eeva et al., 2008). Moreover, because moult can begin early in urban habitats and overlap with late second broods (Hutton et al., 2021), individuals might produce new feathers in or near their breeding territories, and the moult environment might match the breeding environment. (iii) Competitive sorting: as colouration might correlate with competitive ability or condition (García‐Navas et al., 2012; Hegyi et al., 2007; Hill, 1991; Senar, 2006), more chromatic individuals may secure higher‐quality territories, whereas paler individuals are relegated to more impervious, resource‐poor areas. (iv) Behavioural specialisation: Carotenoid‐based plumage colour might reflect different dietary strategies, with paler birds exploiting anthropogenic foods more efficiently and therefore selecting urbanised habitats. In line with this, paler birds in cities have been reported to achieve higher reproductive success than more chromatic birds (Bekka et al., 2025), which is proposed to be associated with a more diverse diet. Paler birds might target a broader range of food sources including ones associated with higher degree of urbanisation, therefore investing less in foraging efforts and competition to access food sources at breeding time. Paler birds might be more adapted to more urbanised places, even within the city, as colouration might reflect their dietary behaviours or other correlated adaptative traits to urbanisation. Disentangling these mechanisms will require longitudinal tracking across breeding and moult, dietary assays (e.g. metabarcoding or stable isotopes), and within‐individual analyses following territory switches or targeted supplementation at moult.

The consistent negative slope across independent cities suggests parallel eco‐evolutionary processes in European great tits, arising from shared plastic responses to urbanisation and/or convergent selection on colouration and its correlates. Given that reproductive performance may covary with colouration in urban habitats (Bekka et al., 2025) and that divergence in carotenoid saturation seems to be driven by genetic changes, or early maternal investments (Biard et al., 2005, 2007; Sandmeyer et al., 2026), a key next step is to quantify within‐city selection gradients on carotenoid‐based colouration across the imperviousness gradient, and test whether the strength, shape and sign of natural selection on carotenoid pigment deposition in feathers are predicted by urban structure (e.g. tree cover, feeder density, pollutant profiles, heat‐island intensity). Nonetheless, this continent‐wide pattern is consistent with the view that cities might serve as replicated, real‐time laboratories for studying eco‐evolutionary dynamics and adaptation (Rivkin et al., 2019; Verrelli et al., 2022).

Because carotenoid‐based colouration covaries with the proportion of impervious surface at territory scale, chroma appears sensitive to local variation in urban habitat structure. This makes carotenoid‐based colouration informative for understanding phenotypic responses to urbanisation processes, with potential applications in planning and monitoring as it may help guide future monitoring towards the specific habitat features most relevant to urban wildlife responses. However, because highly impervious territories are under‐represented in our samples, extrapolation to the most built‐up urban cores is uncertain and range restriction likely attenuates the estimated slope (i.e. a bias towards zero). Finally, we encourage future work to adopt continuous urbanisation metrics rather than urban–rural dichotomies (Szulkin et al., 2020) and to extend long‐term monitoring into highly urbanised and polluted sites. In dichotomous designs, birds labelled ‘urban’ may in fact occupy vegetated fragments within city limits rather than truly hardscaped cores, potentially obscuring continuous responses to urbanisation. While sampling intensely altered areas is challenging, explicitly including these habitats is essential to avoid underestimating effects of urbanisation on wildlife.

## AUTHOR CONTRIBUTIONS

Nicolas Bekka conceived the working hypothesis behind this manuscript, conceived the impervious surfaces cover protocol, validated the data, analysed the data and led the writing of the manuscript. Lisa Sandmeyer contributed to the development of the working hypothesis and to manuscript preparation, providing substantive input during review and editing. Nitha Chardine performed the impervious surfaces cover classification under the supervision and with the help of Nicolas Bekka. Nicolas Bekka performed spectrophotometry for Paris, Harjavalta and Malmö, Andrea S. Grunst and Melissa L. Grunst for Antwerp, Katarzyna Janas for Warsaw and Lisa Sandmeyer for Montpellier. Nicolas Bekka, Clotilde Biard, Nitha Chardine, Marcel Eens, Tapio Eeva, Arnaud Gregoire, Andrea S. Grunst, Melissa L. Grunst, Caroline Isaksson, Katarzyna Janas, Pablo Salmón, Lisa Sandmeyer and Marta Szulkin participated and/or supervised data collection and general monitoring of great tit populations. All authors contributed significantly to the drafts and gave final approval for publication.

## CONFLICT OF INTEREST STATEMENT

The authors declare no conflict of interest.

## Supporting information


**Figure S1.** Example of the two‐class habitat map used to quantify imperviousness (Jardin des Plantes, one of the urban sites in Paris, France). Within each 100‐m buffer (semi‐transparent circles), pixels were classified as vegetation (green: trees, lawns, shrubs) or impervious (light grey: buildings, roads, paved paths) from sub‐meter Google imagery using the Semi‐Automatic Classification Plugin (supervised nearest‐neighbour; ≥10 training polygons per class). Mislabelled roads/buildings were corrected with OpenStreetMap layers (see Section 2). Imagery © Google.
**Table S1.** Sample sizes.
**Table S2.** Continental mixed‐effects model for carotenoid chroma.
**Table S3.** Continental mixed‐effects model for Brightness.
**Table S4.** Continental mixed‐effects model for UV chroma.
**Table S5.** Details for leave‐one‐city‐out analysis.

## Data Availability

The dataset and scripts supporting this study's findings are available in the following Zenodo Repository: https://doi.org/10.5281/zenodo.20058693 (Bekka et al., 2026).
