# Supplementary material for: Paler within the city: Carotenoid‐based colouration covaries negatively with the proportion of impervious surface in great tit (Parus major) territories across Europe
Source: J Anim Ecol. 2026 Sep 18;95(11):2200–13. doi: 10.1111/1365-2656.70355 (PMC13731826; doi:10.1111/1365-2656.70355)
Supplement: Supplementary file 1 [file JANE-95-2200-s001.docx]

**Paler within the city: carotenoid-based colouration covaries negatively with the proportion of impervious surface in great tit (*Parus major*) territories across Europe**

**SUPPLEMENTARY MATERIAL**

**Figure S1.** Example of the two-class habitat map used to quantify imperviousness (Jardin des Plantes, one of the urban sites in Paris, France). Within each 100-m buffer (semi-transparent circles), pixels were classified as vegetation (green: trees, lawns, shrubs) or impervious (light grey: buildings, roads, paved paths) from sub-meter Google imagery using the Semi-Automatic Classification Plugin (supervised nearest-neighbour; ≥10 training polygons per class). Mislabelled roads/buildings were corrected with OpenStreetMap layers (see Methods). Imagery © Google.

**Table S1. Sample sizes**

Sample sizes by study system, sampling year, sex and age class for adult great tits included in the colour analyses. Age was classified as 1-year-old or ≥2-year-old following plumage criteria; individuals with missing age information are indicated as unknown.

| **City** | **Country** | **Sampling**  **Year** | **Total Obs.** | **Males** | **Females** | **1-year-old** | **≥2-year-old** | **Unknown Age** |
| --- | --- | --- | --- | --- | --- | --- | --- | --- |
| **Antwerp** | Belgium | 2017  2018 | 386 | 209 | 177 | 155 | 231 | 0 |
| **Montpellier** | France | 2023 | 141 | 64 | 77 | 50 | 91 | 0 |
| **Paris** | France | 2022  2023  2024 | 110 | 43 | 67 | 46 | 64 | 0 |
| **Harjavalta** | Finland | 2005  2008 | 94 | 0 | 94 | 49 | 36 | 9 |
| **Malmö** | Sweden | 2015 | 60 | 27 | 33 | 21 | 39 | 0 |
| **Warsaw** | Poland | 2023 | 47 | 25 | 22 | 10 | 37 | 0 |
| **Total** | - | - | 838 | 368 | 470 | 331 | 498 | 9 |

**Table S2. Continental mixed-effects model for carotenoid chroma.**

Fixed effects (top) are from the model described in the results section. Estimates are unstandardised coefficients; SE = standard error; df = Satterthwaite degrees of freedom; *t* and *P* test whether the coefficient differs from 0; CIs are Wald 95% intervals. “Imperviousness” is the proportion of impervious surface within 100 m of the nest (0–1 scale; a 0.10 increase corresponds to ~0.005 lower chroma given β ≈ −0.053). Sex is coded with females as the reference; Age is coded with 1-year as reference. Random-effects (bottom) give variance components for City, Year, Individual ID, and the residual.

| **Term** | **Estimate** | **S.E.** | **df** | **t** | **P** | **CI low** | **CI high** |
| --- | --- | --- | --- | --- | --- | --- | --- |
| (Intercept) | 0.400 | 0.048 | 7.336 | 8.290 | < 0.001 | 0.305 | 0.495 |
| **Imperviousness** | -0.053 | 0.020 | 739.134 | -2.649 | < 0.01 | -0.091 | -0.014 |
| **Sex**  *(Female as reference)* | 0.012 | 0.007 | 676.176 | 1.661 | < 0.1 | -0.002 | 0.026 |
| **Age**  *(Age 1 as reference)* | 0.042 | 0.007 | 813.895 | 6.117 | < 0.001 | 0.028 | 0.055 |
| **Random Effects** | | | | | | | |
|  | | Variance | Std. Dev. |  | | | |
| City | | 0.010 | 0.100 |  |  |  |  |
| Individual ID | | 0.004 | 0.065 |  |  |  |  |
| Year | | 0.003 | 0.059 |  |  |  |  |
| Residual | | 0.005 | 0.068 |  |  |  |  |

**Table S3. Continental mixed-effects model for Brightness.**

Fixed effects (top) are from the model exploring total brightness variation. Estimates are unstandardised coefficients; SE = standard error; df = Satterthwaite degrees of freedom; *t* and *P* test whether the coefficient differs from 0; CIs are Wald 95% intervals. “Imperviousness” is the proportion of impervious surface within 100 m of the nest (0–1 scale). Sex is coded with females as the reference; Age is coded with 1-year-old as reference. Random-effects (bottom) give variance components for City, Year, Individual ID, and the residual.

| **Term** | **Estimate** | **S.E.** | **df** | **t** | **P** | **CI low** | **CI high** |
| --- | --- | --- | --- | --- | --- | --- | --- |
| (Intercept) | 11131.365 | 1235.673 | 6.707 | 9.008 | < 0.001 | 8709.490 | 13553.240 |
| **Imperviousness** | 242.606 | 438.910 | 789.767 | 0.553 | 0.58 | -617.642 | 1102.854 |
| **Sex**  *(Female as reference)* | 1141.859 | 158.967 | 765.942 | 7.183 | < 0.001 | 830.290 | 1453.428 |
| **Age**  *(Age 1 as reference)* | -10.864 | 153.334 | 816.173 | -0.071 | 0.94 | -311.393 | 289.6655 |
| **Random Effects** | | | | | | | |
|  | | Variance | Std. Dev. |  | | | |
| City | | 6442163 | 2538 |  |  |  |  |
| Individual ID | | 1208714 | 1099 |  |  |  |  |
| Year | | 2668737 | 1634 |  |  |  |  |
| Residual | | 3216324 | 1793 |  |  |  |  |

**Table S4. Continental mixed-effects model for UV chroma.**

Fixed effects (top) are from the model exploring UV chroma variation. Estimates are unstandardised coefficients; SE = standard error; df = Satterthwaite degrees of freedom; *t* and *P* test whether the coefficient differs from 0; CIs are Wald 95% intervals. “Imperviousness” is the proportion of impervious surface within 100 m of the nest (0–1 scale). Sex is coded with females as the reference; Age is coded with 1-year-old as reference. Random-effects (bottom) give variance components for City, Year, Individual ID, and the residual.

| **Term** | **Estimate** | **S.E.** | **df** | **t** | **P** | **CI low** | **CI high** |
| --- | --- | --- | --- | --- | --- | --- | --- |
| (Intercept) | 0.229 | 0.013 | 8.661 | 17.315 | < 0.001 | 0.198 | 0.248 |
| **Imperviousness** | 0.0005 | 0.006 | 655.4 | 0.082 | 0.93 | -0.011 | 0.012 |
| **Sex**  *(Female as reference)* | 0.003 | 0.002 | 773.7 | 1.388 | 0.17 | -0.001 | 0.007 |
| **Age**  *(Age 1 as reference)* | 0.001 | 0.002 | 818.0 | 0.417 | 0.68 | -0.003 | 0.005 |
| **Random Effects** | | | | | | | |
|  | | Variance | Std. Dev. |  | | | |
| City | | 0.000 | 0.005 |  |  |  |  |
| Individual ID | | 0.000 | 0.016 |  |  |  |  |
| Year | | 0.001 | 0.034 |  |  |  |  |
| Residual | | 0.001 | 0.024 |  |  |  |  |

**Table S5. Details for leave-one-city-out analysis.**

For each refit of the continental model, we dropped one city and re-estimated the fixed effect of imperviousness. Rows report the slope for imperviousness, its SE, and Wald 95% CI; the first row (“Full data”) shows the estimate using all cities. Negative values indicate lower carotenoid chroma with higher impervious cover.

| **Dropped** | **Slope**  **Estimate** | **S.E.** | **CI low** | **CI high** |
| --- | --- | --- | --- | --- |
| (Full dataset) | -0.053 | 0.020 | -0.091 | -0.014 |
| **Antwerp** | -0.061 | 0.023 | -0.111 | -0.011 |
| **Harjavalta** | -0.063 | 0.022 | -0.106 | -0.020 |
| **Malmö** | -0.055 | 0.020 | -0.096 | -0.015 |
| **Montpellier** | -0.032 | 0.022 | -0.075 | 0.011 |
| **Paris** | -0.04 | 0.021 | -0.085 | -0.003 |
| **Warsaw** | -0.054 | 0.021 | -0.095 | -0.013 |
